# Immune escape in portal vein tumor thrombus: an intravascular tumor–immune niche framework for hepatocellular carcinoma

**DOI:** 10.3389/fimmu.2026.1946103

**Published:** 2026-09-16

**Authors:** Xingfei Li, Delin Ma, Zhigao Yuan, Jiye Zhu, Jie Gao

**Affiliations:** 1 Department of Hepatobiliary Surgery, Peking University People’s Hospital, Beijing, China; 2 Peking University Research Center for Diagnosis & Treatment of Liver Cancer, Beijing, China; 3 Peking University Institute of Organ Transplantation, Beijing, China

**Keywords:** cancer immunotherapy, hepatocellular carcinoma, immune escape, portal vein tumor thrombus, spatial immunology, tumor–immune microenvironment

## Abstract

Portal vein tumor thrombus (PVTT) is a major manifestation of macrovascular invasion in hepatocellular carcinoma (HCC) and is associated with aggressive progression and heterogeneous responses to immune checkpoint inhibitor–based therapy. Current stratification relies mainly on anatomical extent, liver function, and tumor burden and does not fully capture the biology of the intravascular lesion. In this Review, we propose PVTT as an anatomically distinct, lesion-centered intravascular tumor–immune niche for hypothesis generation rather than as an established autonomous immune compartment. We distinguish PVTT-direct evidence from supportive venous-thrombus/metastatic evidence and HCC-extrapolated mechanisms. Current PVTT-direct evidence is strongest for clonal and spatial divergence and PVTT-specific stromal remodeling, including myofibroblast-like cancer-associated fibroblast accumulation, NID1-associated immune barriers, and FAP-positive fibroblast/GJA5-positive endothelial hubs. Venous-thrombus studies provide supportive evidence for macrophage-associated immune suppression, including C5aR-positive tumor-associated macrophages, whereas hypoxia–adenosine signaling, adaptive checkpoint activation, and lipid metabolic rewiring remain largely extrapolated candidate mechanisms requiring direct PVTT validation. On this basis, we organize the available evidence into literature-informed candidate resistance phenotypes and outline a translational framework integrating paired primary tumor–PVTT sampling, spatial and single-cell profiling, functional imaging, and liquid biopsy. These phenotypes are conceptual groupings rather than empirically derived or validated patient subtypes and should guide mechanistic testing and biomarker-enriched trial design rather than routine treatment assignment. Prospective PVTT-specific studies with anatomically resolved sampling, longitudinal pharmacodynamic assessment, and biomarker-by-treatment interaction analyses are required to determine whether this lesion-centered niche framework can ultimately provide clinically useful stratification.

## 1 Introduction

Portal vein tumor thrombus (PVTT), a manifestation of macrovascular invasion, represents one of the most aggressive and treatment-refractory forms of hepatocellular carcinoma (HCC). It is associated with rapid intrahepatic dissemination, limited treatment options, and poor prognosis (1–3). Although immune checkpoint inhibitor (ICI)-based combinations have reshaped the treatment landscape of advanced HCC, patients with extensive PVTT, particularly main-trunk involvement, remain underrepresented in pivotal trials and incompletely addressed by current algorithms (2, 4–7). Treatment selection therefore still depends largely on anatomical extent, liver reserve, tumor burden, performance status, portal hypertension, and bleeding risk.

These clinical variables do not fully capture the immune, stromal, myeloid, metabolic, and dissemination programs that may contribute to heterogeneous responses. Single-cell and paired-tissue studies suggest that venous tumor thrombi can contain immune and molecular states that differ from those of the primary tumor, while integrated analyses have identified intensified endothelial–fibroblast–myeloid communication in combined metastatic compartments (8–11). More recent PVTT-specific spatial multi-omics studies have directly identified myofibroblast-like cancer-associated fibroblast (myCAF) accumulation, NID1-associated immune barriers, and FAP-positive fibroblast/GJA5-positive endothelial stromal hubs, strengthening the evidence for site-specific immune–stromal remodeling (12, 13). These observations support a niche-based interpretation of PVTT biology, but they do not establish PVTT as a uniform or completely immune-isolated compartment.

We therefore use “intravascular tumor–immune niche” as a cautious, hypothesis-generating framework. The related term “immune sanctuary” is used only as shorthand for locally constrained antitumor immunity that may be difficult to restore, not as an established pathological classification. This designation does not imply established treatment resistance or treatment-predictive utility. This Review focuses on how site-dependent evolution, immune compartmentalization, stromal–myeloid barriers, metabolic stress, and potential links to systemic dissemination can be translated into testable biomarkers and mechanism-informed clinical studies. Unlike treatment-centered summaries, the synthesis is organized according to the provenance and strength of evidence. Accordingly, this Review adopts a PVTT-centered, lesion-specific perspective, using anatomical context and evidence provenance as the organizing principles for interpreting immune escape, candidate resistance phenotypes, and translational opportunities.

The conceptual novelty of this framework is not to assert that PVTT is already established as an autonomous immune compartment, but to treat it as an anatomically distinct lesion in which local vascular context, clonal evolution, and immune–stromal remodeling may generate site-dependent biology that should be evaluated separately from the primary tumor. Thus, the term “niche” denotes a falsifiable, lesion-centered working model rather than a proven PVTT-specific immune entity (**Figure 1**).

**Figure 1:**
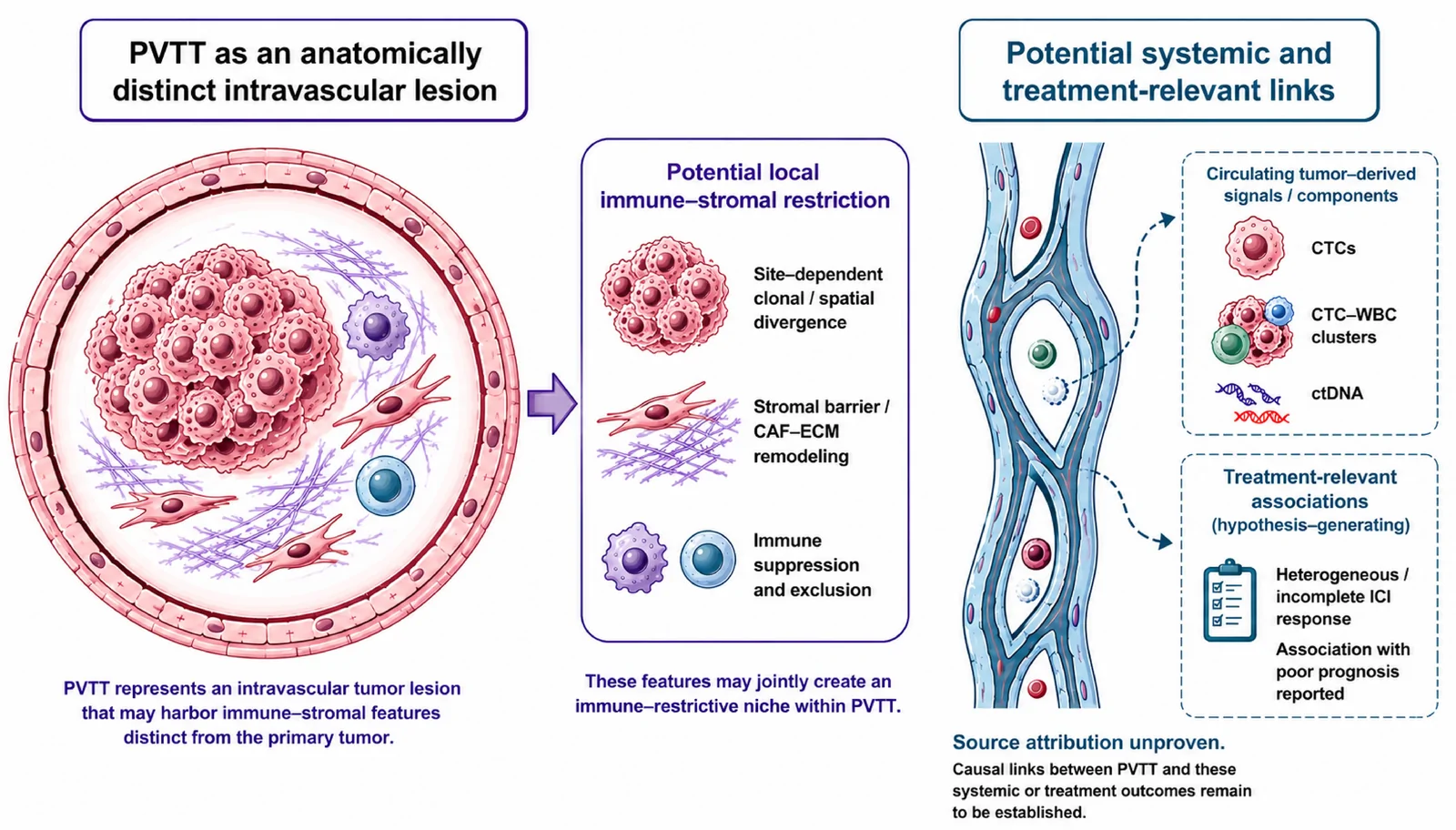
Conceptual architecture of the proposed PVTT intravascular tumor–immune niche. This schematic presents PVTT as an anatomically distinct intravascular lesion and a lesion-centered, hypothesis-generating working model rather than an established autonomous immune compartment. The proposed local niche is organized around site-dependent clonal/spatial divergence, stromal/ECM remodeling, and immune suppression or exclusion, with the strength and maturity of evidence differing across these components. Circulating tumor-derived signals, including CTCs, CTC–WBC clusters, and ctDNA, together with reported associations with heterogeneous ICI response and poor prognosis, are shown as potential systemic and treatment-relevant links. Direct source attribution, causal treatment resistance, and treatment-predictive value remain unproven. CAF, cancer-associated fibroblast; CTC, circulating tumor cell; ctDNA, circulating tumor DNA; ECM, extracellular matrix; ICI, immune checkpoint inhibitor; PVTT, portal vein tumor thrombus; WBC, white blood cell.

## 2 Review scope, literature search, and evidence framework

This is a narrative, mechanism-oriented review rather than a systematic review or meta-analysis. A targeted search of PubMed/MEDLINE, Embase, and Web of Science was conducted through July 20, 2026, using combinations of terms related to “hepatocellular carcinoma/HCC,” “portal vein tumor thrombus/PVTT,” “venous tumor thrombus,” “tumor immune microenvironment,” “immunotherapy,” “single-cell,” “spatial transcriptomics,” “multiplex imaging,” and “liquid biopsy.” Reference lists of relevant original studies and reviews were also screened. Priority was given to studies using anatomically confirmed PVTT specimens, paired primary tumor–PVTT samples, or PVTT-specific analyses with separately reported results. Studies combining PVTT with other venous thrombi or metastatic sites were not considered PVTT-specific unless the PVTT component could be analyzed separately. Dedicated PVTT imaging and clinical cohorts were also prioritized.

Evidence was categorized as PVTT-direct, supportive venous-thrombus/metastatic, or HCC-extrapolated. Direct evidence was derived from anatomically confirmed PVTT specimens or PVTT-specific analyses, whereas studies without separable PVTT data were classified as supportive or extrapolated evidence and were used primarily to nominate candidate mechanisms rather than establish PVTT-specific causality or predictive value. Separately from evidence provenance, we distinguish three levels of evidentiary maturity: established clinical context, emerging PVTT-specific biological evidence, and hypothesis-generating mechanisms requiring validation. Established evidence refers primarily to anatomical classification, prognosis, and treatment outcomes supported by clinical studies. Emerging evidence includes reproducible but still limited PVTT-specific molecular, cellular, or spatial observations. Hypothesis-generating mechanisms are those supported mainly by indirect, extrapolated, preclinical, or insufficiently replicated data and are presented as priorities for future testing rather than as established features of PVTT biology.

Within this framework, we distinguish evidence for spatial compartmentalization, functional immune restriction, treatment resistance, and treatment-predictive value.

Spatial compartmentalization refers to reproducible differences in immune or stromal organization between PVTT and matched or anatomically relevant comparator tissue.

Functional immune restriction requires evidence linking these features to impaired effector-cell activity or active suppressive interactions. Associations with treatment resistance are considered downstream clinical correlates and do not by themselves establish an immune-restricted niche. Treatment-predictive value requires biomarker-by-treatment interaction and represents a higher level of translational validation rather than a defining criterion. Accordingly, “immune-restricted niche” is used here as a working designation when spatially resolved and functional evidence converge.

The principal PVTT-focused biological, biomarker, imaging, and clinical treatment studiesunderlying this synthesis are summarized at the study level in **Supplementary Table S1**, which reports study design and population, sample size, specimens or intervention, experimental approach or clinical endpoints, PVTT classification when reported, major findings or outcomes, and the current evidence status or biomarker role.

## 3 Clinical heterogeneity and the need for biological stratification

PVTT is commonly classified using the Japanese Vp system or the Chinese Cheng classification. In the Vp system, Vp3 denotes tumor thrombus involving a first-order portal vein branch, whereas Vp4 denotes involvement of the main portal vein trunk and/or a contralateral portal vein branch. In the Cheng classification, types I–IV represent progressively greater anatomical extent, from segmental or sectoral portal vein branches to the right/left portal vein, main portal vein, and superior mesenteric vein, respectively. The 2026 Chinese guideline recommends the Cheng classification as the national standard and emphasizes multidisciplinary, individualized management according to liver function, tumor resectability, and PVTT type (3). More extensive PVTT is generally associated with higher tumor burden, intrahepatic dissemination, impaired liver function, fewer therapeutic options, and shorter survival (1–3). However, patients within the same anatomical category may differ substantially in immune-cell infiltration, spatial exclusion, macrophage programs, hypoxia, metabolic activity, circulating tumor-cell burden, and sensitivity to ICI-based therapy. Anatomical staging should therefore remain the clinical foundation but be complemented, in research settings, by tissue, spatial, imaging, and circulating biomarkers that capture distinct dimensions of PVTT biology.

Against this established clinical context, Section 4 distinguishes emerging PVTT-specific biological findings from hypothesis-generating mechanisms that remain to be validated.

## 4 Biological basis and boundaries of the proposed PVTT tumor–immune niche

Immunotherapy resistance in HCC-PVTT is unlikely to be explained by a single checkpoint or by tumor burden alone. Conceptually, the framework is organized hierarchically from anatomical context and PVTT-specific biological evidence, through local immune–stromal mechanisms, to candidate resistance phenotypes and their translational implications. The biological basis for a PVTT-centered niche is currently strongest for site-dependent clonal or ecosystem divergence and PVTT-specific stromal organization. Myeloid suppression is supported mainly by venous-thrombus or metastatic evidence, whereas hypoxia–adenosine signaling, adaptive checkpoint activation, and lipid metabolic remodeling remain predominantly HCC-extrapolated candidate mechanisms. Accordingly, the following sections distinguish features that currently support the niche concept from mechanisms included primarily for hypothesis generation. The proposed framework comprises five interconnected dimensions: clonal and spatial divergence; effector T-cell dysfunction and myeloid suppression; stromal, vascular, and metabolic barriers; potential links to systemic dissemination; and adaptive resistance under therapeutic pressure (4, 9, 11, 14, 15) (**Figure 2**).

**Figure 2:**
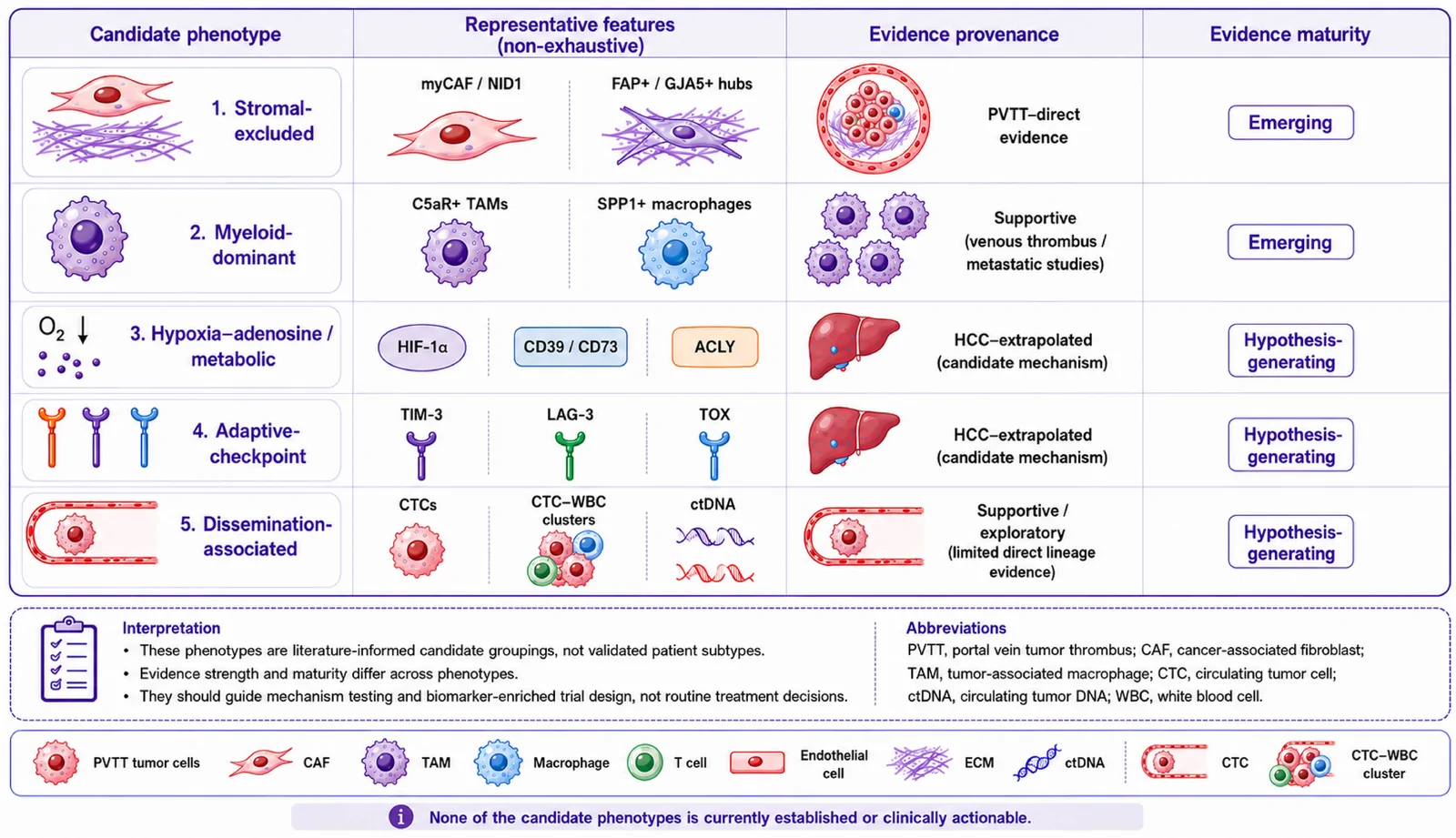
Candidate resistance phenotypes and evidence provenance within the proposed PVTT niche. This matrix summarizes five literature-informed candidate resistance phenotypes—stromal-excluded, myeloid-dominant, hypoxia–adenosine/metabolic, adaptive-checkpoint, and dissemination-associated—as conceptual groupings rather than empirically derived or validated patient subtypes. Representative features are shown together with evidence provenance (PVTT-direct, supportive venous-thrombus/metastatic, or HCC-extrapolated) and evidentiary maturity (emerging or hypothesis-generating). PVTT-direct evidence is strongest for stromal programs; myeloid programs are supported mainly by venous-thrombus/metastatic studies; hypoxia–adenosine/metabolic and adaptive-checkpoint programs remain predominantly HCC-extrapolated; and dissemination-associated signals remain supportive or exploratory, with direct PVTT-source attribution unproven. None of these candidate phenotypes is currently established, clinically actionable, or validated for treatment selection. ACLY, ATP citrate lyase; C5aR, complement component 5a receptor; CAF, cancer-associated fibroblast; CD39, ectonucleoside triphosphate diphosphohydrolase 1; CD73, ecto-5′-nucleotidase; CTC, circulating tumor cell; ctDNA, circulating tumor DNA; ECM, extracellular matrix; FAP, fibroblast activation protein; GJA5, gap junction protein alpha 5; HCC, hepatocellular carcinoma; HIF-1α, hypoxia-inducible factor 1 alpha; LAG-3, lymphocyte-activation gene 3; myCAF, myofibroblast-like cancer-associated fibroblast; NID1, nidogen 1; PVTT, portal vein tumor thrombus; SPP1, secreted phosphoprotein 1; TAM, tumor-associated macrophage; TIM-3, T-cell immunoglobulin and mucin-domain containing 3; TOX, thymocyte selection-associated high mobility group box; WBC, white blood cell.

### 4.1 Clonal and spatial divergence between primary HCC and PVTT

PVTT develops within the portal venous system and is therefore a spatially distinct intravascular lesion. Paired genomic analysis indicates that primary HCC and PVTT may share a common origin but subsequently follow divergent trajectories, with thrombus-clonal alterations reported in individual cases (9). Single-cell atlases likewise support site-dependent ecosystem divergence between primary tumors and venous tumor thrombi (8, 11). Supportive venous-thrombus/metastatic evidence from a larger analysis integrating primary HCC, PVTT, and metastatic lymph nodes identified expansion of SPP1-positive and TREM2-positive macrophage programs and intensified endothelial–fibroblast–myeloid communication in the combined metastatic compartment; however, the pooled analysis precludes PVTT-specific attribution (10).

Spatial immune organization remains incompletely defined but is no longer supported only by extrapolation from primary HCC. A paired multi-omics study of 99 specimens from 47 patients generated a spatial map of PVTT and identified myCAF enrichment arising through macrophage-to-myofibroblast transition, with NID1 concentrated in PVTT cores and linked to immune-barrier formation (12). A second study integrating single-cell transcriptomics, chromatin accessibility, and spatial transcriptomics identified FAP-positive fibroblasts and GJA5-positive arterial endothelial cells as central stromal hubs spatially associated with hypoxia–epithelial–mesenchymal-transition tumor cells, and functionally implicated SLIT2/ROBO1 signaling in metastatic progression (13). These studies provide direct evidence for PVTT-specific stromal organization, although external validation, standardized spatial scoring, and treatment-predictive evidence remain lacking. The prevalence and functional significance of tertiary lymphoid structures within PVTT still require direct validation (16). More broadly, primary-HCC studies indicate that fibroblast recruitment and spatial tissue architecture can contribute to local immune–stromal organization (17, 18), although these observations do not provide direct evidence for TLS or immune restriction within PVTT. These findings represent emerging PVTT-specific biological evidence rather than an established universal feature.

### 4.2 Effector T-cell dysfunction and myeloid suppression

Supportive venous-thrombus evidence implicates macrophage-associated suppression of effector T cells. C5aR-positive tumor-associated macrophages are enriched in venous tumor thrombi and can suppress granzyme B expression in CD8-positive T cells (11). Other thrombus-associated or combined-site datasets nominate MMP9/SPP1 and SPP1/TREM2 macrophage states that may interact with stromal and endothelial programs (8, 10). These findings position myeloid suppression, rather than checkpoint expression alone, as a central candidate mechanism of local immune dysfunction.

Several additional pathways remain less directly supported. CCL2/CCR2 signaling recruits monocyte-derived macrophages in primary HCC, while TAM receptor tyrosine kinases and macrophage CD39–adenosine signaling have been linked to tumor progression or anti-PD-1 resistance in broader HCC models (14, 19, 20). C5aR-positive TAMs have supportive venous-thrombus evidence, whereas CCL2/CCR2, TAM-receptor, and adenosine-related macrophage programs remain HCC-extrapolated candidate mechanisms until anatomically resolved PVTT studies are available. This represents supportive/emerging evidence, with PVTT-specific functional validation still incomplete.

### 4.3 Stromal, vascular, and metabolic barriers

Cancer-associated fibroblasts (CAFs), extracellular matrix (ECM), and dysfunctional tumor endothelium are plausible contributors to spatial immune exclusion. CAF-mediated matrix deposition can increase tissue stiffness and restrict CD8-positive T-cell infiltration, while CAF–endothelial interactions can impair vascular organization and immune-cell extravasation (21–24). Recent PVTT-specific multi-omics studies have identified myCAF enrichment, macrophage-to-myofibroblast transition, NID1-rich immune barriers in thrombus cores, and FAP-positive fibroblast/GJA5-positive endothelial hubs (12, 13). Primary-HCC studies additionally implicate fibroblast-associated signaling, including tumor-derived Egfl7, in metastatic remodeling and immune exclusion (17, 18). Collectively, these data support PVTT-specific stromal remodeling, but they do not yet establish a universally reproducible fibroblast taxonomy, standardized spatial cutoff, or validated treatment-predictive biomarker.

Direct measurements of oxygen tension and metabolites in PVTT are scarce. HCC-extrapolated evidence implicates macrophage CD39–adenosine activity, ACLY-dependent lipogenesis, and fatty-acid handling in immunosuppression or tumor fitness (14, 25–27). HIF-1α, CD39/CD73, ACLY, and fatty-acid transport programs should therefore be treated as HCC-extrapolated candidates until validated in anatomically annotated PVTT tissue.

The stromal, vascular, myeloid, and metabolic compartments are likely interdependent: matrix remodeling can worsen immune exclusion and hypoxia; hypoxia can reinforce suppressive myeloid programs; and adenosine or lipid metabolism can sustain T-cell dysfunction. This network provides a mechanistic rationale for studying combinations that remodel the niche, but current evidence does not support assigning therapy from these markers in routine practice (**Figure 3**). These remain hypothesis-generating, predominantly HCC-extrapolated mechanisms.

**Figure 3:**
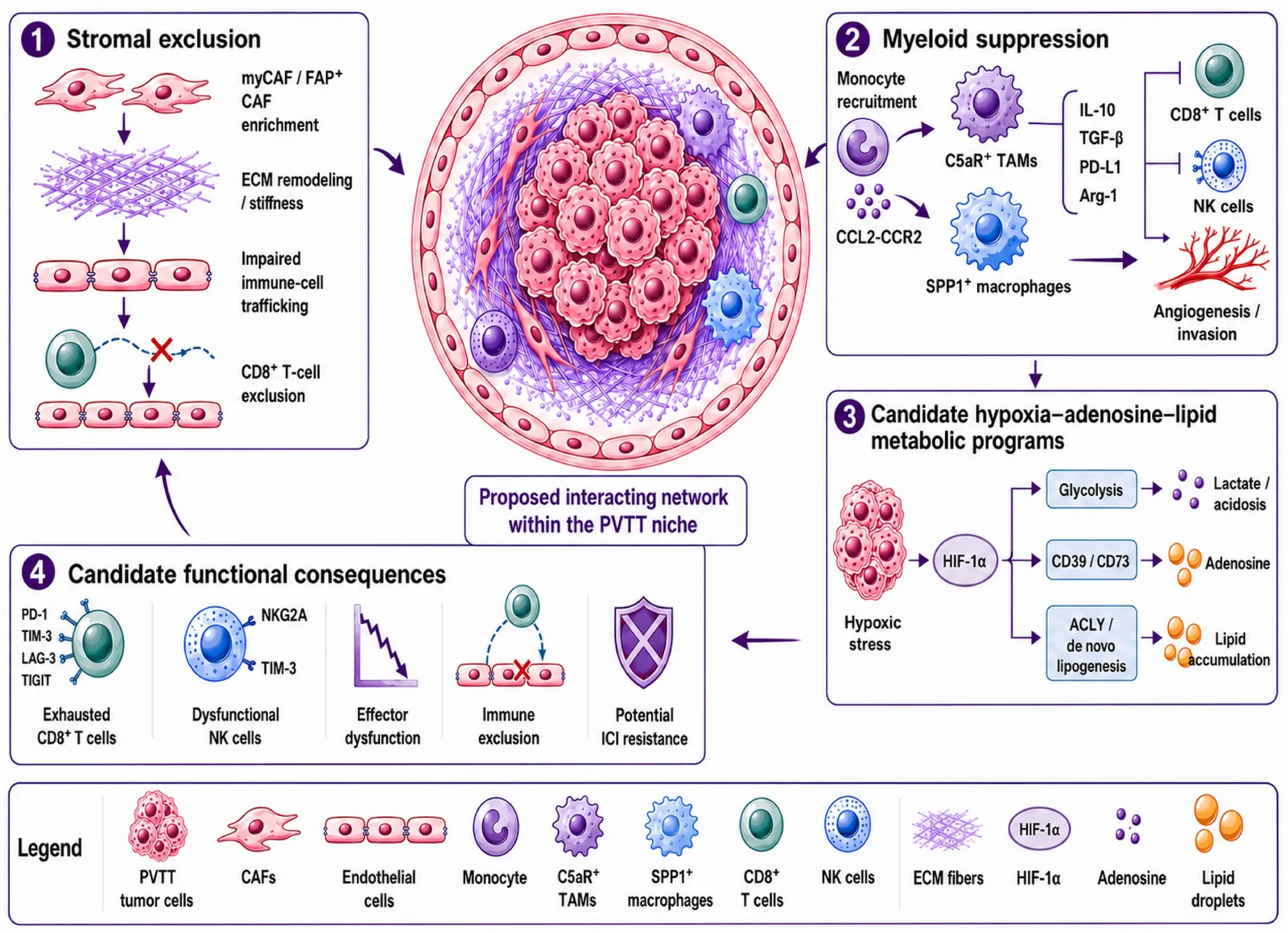
Local stromal–myeloid–metabolic interactions within the proposed PVTT niche. This schematic presents a hypothesis-generating model of local stromal, myeloid, and metabolic interactions that may contribute to immune restriction and potential treatment resistance in PVTT. Emerging PVTT-direct studies support myCAF- and FAP-associated stromal remodeling, whereas the proposed effects of ECM remodeling on immune-cell trafficking are supported mainly by broader HCC and tumor-stroma evidence. Supportive venous-thrombus/metastatic studies implicate C5aR-positive TAMs and SPP1-positive macrophage states, while CCL2/CCR2 signaling, suppressive cytokine programs, and the hypoxia–HIF-1α, CD39/CD73–adenosine, glycolytic, and ACLY-dependent lipid pathways remain predominantly HCC-extrapolated and require direct validation in anatomically resolved PVTT tissue. The depicted functional consequences—including CD8-positive T-cell exhaustion, NK-cell dysfunction, effector dysfunction, immune exclusion, angiogenic or invasive behavior, and potential ICI resistance—are candidate outcomes rather than established PVTT-specific causal mechanisms. Arrows indicate proposed biological relationships rather than validated causal pathways. ACLY, ATP citrate lyase; Arg-1, arginase 1; CAF, cancer-associated fibroblast; C5aR, complement component 5a receptor; CCL2, C-C motif chemokine ligand 2; CCR2, C-C motif chemokine receptor 2; CD39, ectonucleoside triphosphate diphosphohydrolase 1; CD73, ecto-5′-nucleotidase; ECM, extracellular matrix; FAP, fibroblast activation protein; HCC, hepatocellular carcinoma; HIF-1α, hypoxia-inducible factor 1 alpha; ICI, immune checkpoint inhibitor; IL-10, interleukin 10; LAG-3, lymphocyte-activation gene 3; myCAF, myofibroblast-like cancer-associated fibroblast; NK, natural killer; NKG2A, natural killer group 2 member A; PD-1, programmed cell death protein 1; PD-L1, programmed death-ligand 1; PVTT, portal vein tumor thrombus; SPP1, secreted phosphoprotein 1; TAM, tumor-associated macrophage; TGF-β, transforming growth factor beta; TIGIT, T-cell immunoreceptor with immunoglobulin and ITIM domains; TIM-3, T-cell immunoglobulin and mucin-domain containing 3.

### 4.4 Potential systemic links and circulating biomarkers

Because PVTT occupies the portal venous lumen, it may be associated with potential links to systemic dissemination; however, direct evidence that PVTT itself serves as the source of circulating tumor populations is limited. HCC studies have identified immune-evasive circulating tumor-cell (CTC) states, associated a G6PD-positive/CSV-positive CTC phenotype with PVTT formation, and linked CTC–white blood cell clusters to the presence of PVTT (28–30). Paired tissue–blood phylogenetic analyses are required to distinguish thrombus-derived signals from those released by the primary tumor or other lesions.

Peripheral inflammatory indices, CTCs, CTC–white blood cell clusters, circulating tumor DNA (ctDNA), and extracellular-vesicle cargos may provide complementary systemic measurements, but they should not be used as surrogates for the local PVTT immune state (31–35). Likewise, higher 18F-fluorodeoxyglucose uptake within PVTT is associated with adverse outcome and may capture metabolic aggressiveness, but it has not been shown to identify a specific immune mechanism or predict benefit from a particular treatment (36).

### 4.5 On-treatment adaptive immune escape

Serial PVTT-biopsy data during ICI-based therapy are almost absent. In primary HCC and broader cohorts, dysfunctional T-cell states and treatment-associated microenvironmental remodeling have been observed, while suppressive programs may persist (4, 8, 37, 38). Adaptive resistance in PVTT is therefore biologically plausible but remains an unproven dynamic process rather than an established thrombus-specific mechanism.

TIM-3, LAG-3, VISTA, TOX, TAM receptor signaling, CD39/CD73 activity, and metabolic remodeling are candidate pharmacodynamic biomarkers for prospective longitudinal evaluation. No paired pre-treatment and on-treatment PVTT study has demonstrated that compensatory expression of these pathways mediates resistance after PD-1/PD-L1 blockade. Their inclusion in the framework is intended to guide serial sampling and mechanistic testing, not to justify treatment switching or dual-checkpoint therapy outside prospective studies.

## 5 Therapeutic implications of candidate PVTT immune phenotypes

The candidate resistance phenotypes proposed here are literature-informed conceptual groupings based on convergent mechanistic signals across evidence tiers; they were not derived from unsupervised clustering or prospective biomarker-defined patient cohorts and should not be interpreted as validated molecular subtypes. Accordingly, none of these candidate phenotypes is currently clinically actionable or validated for treatment selection, and their potential relevance to treatment stratification requires prospective validation and biomarker-by-treatment interaction analyses. The proposed tumor–immune niche contains potentially targetable vulnerabilities rather than a single dominant checkpoint. The therapeutic implications below are hypotheses for prospective testing: locoregional therapies may provide cytoreduction and potential immune-modulating effects; antiangiogenic agents or tyrosine kinase inhibitors (TKIs) may modify vascular barriers; and investigational macrophage-, stromal-, adenosine-, or metabolism-directed agents may weaken candidate resistance programs. None of the proposed phenotype–treatment links is currently validated for clinical treatment selection.

### 5.1 Locoregional treatment as a candidate immune-modulating strategy

Radiotherapy, hepatic arterial infusion chemotherapy (HAIC), and transarterial chemoembolization (TACE) are used primarily for local control and cytoreduction, but they may also exert candidate immune-modulating effects through changes in antigen release, inflammatory signaling, and the tumor microenvironment (37, 39). This provides a rationale for testing locoregional treatment with ICI-based therapy in patients who require rapid control of high-burden or extensive PVTT. However, the magnitude, timing, and durability of such potential immune-modulating effects within PVTT have not been demonstrated prospectively.

Locoregional approaches should not be considered immunologically equivalent. Radiotherapy may promote tumor-cell injury, antigen release, and inflammatory signaling without inducing arterial ischemia, whereas TACE combines cytotoxic exposure with embolization-induced ischemic necrosis and may simultaneously enhance antigen release and reinforce hypoxia- and VEGF-related suppressive programs. HAIC differs from TACE by delivering high local concentrations of cytotoxic agents through the hepatic artery without intentional embolization, potentially providing rapid cytoreduction and antigen exposure without the same degree of ischemic stress. These modality-specific immune effects remain biologically plausible rather than established in PVTT, because paired pre- and post-treatment immune profiling of PVTT is largely unavailable (37, 39).

Recent PVTT cohorts have evaluated HAIC-, TACE-, and radiotherapy-based combinations with ICIs and/or targeted agents (40–47). Clinical evidence supports the activity of HAIC-based combinations in PVTT, although it does not establish immune priming. In a retrospective cohort of 119 patients receiving HAIC plus anti-PD-1 therapy, with TKIs used in most patients, median overall survival and progression-free survival were 14.9 and 6.9 months, respectively, with an objective response rate of 21.2% (42). In another retrospective study of patients with Vp3/Vp4 PVTT treated with mFOLFOX-based HAIC plus TKIs and ICIs, the PVTT-specific objective response rate was 75.7% and median overall survival was 15.8 months (43).

Similar clinical signals have been reported with other locoregional combinations. In a multicenter retrospective PVTT cohort, TACE plus apatinib and PD-1 blockade was associated with longer median progression-free survival (6.9 vs. 4.0 months) and overall survival (14.6 vs. 8.5 months) and a higher objective response rate (53.6% vs. 17.9%) than TACE plus apatinib after propensity-score matching (45). Radiotherapy-based combinations have likewise shown encouraging activity, with recent PVTT studies reporting improved response and survival with SBRT-containing ICI–antiangiogenic regimens (40, 47).

Importantly, these studies demonstrate clinical activity rather than an established immune-priming mechanism. Their predominantly nonrandomized designs, treatment heterogeneity, and lack of paired pre- and post-treatment PVTT immune profiling or validated pharmacodynamic immune endpoints preclude attribution of clinical benefit to immune modulation. Future trials should incorporate longitudinal tissue sampling and prespecified pharmacodynamic biomarkers to determine whether individual locoregional modalities remodel the PVTT immune microenvironment.

### 5.2 Vascular remodeling with antiangiogenic agents or TKIs

Antiangiogenic therapy is relevant to HCC-PVTT because vascular invasion coexists with abnormal angiogenic and immune programs. Vascular normalization may improve perfusion and immune-cell trafficking, although these effects have not been demonstrated directly in paired PVTT samples (23, 48). The IMbrave150 trial established the clinical activity of atezolizumab plus bevacizumab in unresectable HCC. In the exploratory Vp4 subgroup, median overall survival was 7.6 months with atezolizumab plus bevacizumab versus 5.5 months with sorafenib, while median progression-free survival was 5.4 versus 2.8 months, respectively (49, 50). These findings support clinical activity in extensive PVTT but remain exploratory and do not establish a vascular or immune biomarker for treatment selection.

TKI–ICI combinations provide an alternative when bevacizumab is unsuitable or broader kinase inhibition is desired (51). However, no validated vascular or hypoxia biomarker currently selects between anti-VEGF antibody-based and TKI-based regimens in PVTT. Treatment decisions must continue to account for liver reserve, portal hypertension, varices, bleeding history, tumor burden, and expected toxicity rather than presumed biomarker superiority (2, 3).

### 5.3 Myeloid- and stromal-directed opportunities

Supportive thrombus-linked evidence, particularly the enrichment and functional activity of C5aR-positive TAMs (11), makes macrophage-directed intervention a biologically plausible research strategy. Candidate approaches include C5aR, CCL2/CCR2, CSF-1/CSF-1R, and TAM-receptor pathways, but their activity and predictive biomarkers remain unvalidated in HCC-PVTT (11, 19, 52, 53). Future studies should establish which macrophage states are reproducible across centers, spatially linked to effector-cell dysfunction, pharmacodynamically modifiable, and associated with differential treatment benefit.

CAF/ECM-directed approaches now have direct PVTT-specific mechanistic support, although clinical translation remains preliminary. In the NID1 study, repurposed acarbose disrupted myCAF-mediated immune barriers, suppressed PVTT progression, and enhanced anti-PD-1 activity in preclinical models; the lower PVTT incidence observed among acarbose-treated patients in an independent cohort remains hypothesis-generating rather than proof of therapeutic efficacy (12). The FAP/GJA5 study identified stromal–tumor crosstalk, including the SLIT2/ROBO1 axis, as an additional candidate vulnerability (13). Because fibroblasts and endothelial cells are heterogeneous and can support tissue homeostasis, selective modulation of pathogenic subsets or signaling programs is preferable to nonspecific stromal depletion. No stromal biomarker is currently validated for treatment selection; prospective pharmacodynamic studies, standardized spatial assays, safety evaluation, and biomarker-by-treatment interaction testing are required.

### 5.4 Adenosine- and metabolism-directed opportunities

Adenosine and lipid metabolism are biologically plausible but predominantly HCC-extrapolated resistance pathways. Macrophage CD39 activity, CD73, A2A/A2B receptors, ACLY, and fatty-acid transport signatures may nominate biomarker-enriched experimental cohorts (14, 25–27). These interventions should be tested with predefined pharmacodynamic endpoints, PVTT-specific expression data, and careful toxicity assessment rather than assumed to be effective on the basis of primary-HCC biology.

Overall, candidate therapeutic implications should be framed as trial-design hypotheses. FDG-avid or high-burden PVTT may motivate studies of locoregional intensification; vascularly abnormal disease may motivate antiangiogenic combinations; and myeloid- or stromal-enriched lesions may motivate macrophage- or CAF-directed trials. Prospective validation must precede any phenotype-based treatment rule (**Table 1**).

**Table 1:** Candidate immune-resistance phenotypes and validation priorities in HCC-PVTT.

Candidate phenotype	Candidate biomarker panel	Evidence tier and source	Detection platform	Candidate therapeutic implication	Priority validation need
Stromal-excluded	myCAF/FAP signatures; NID1; GJA5-positive endothelial programs; collagen organization; spatial T-cell exclusion	PVTT-direct: myCAF/MMT–NID1 immune barriers and FAP/GJA5 stromal hubs (12, 13); broader CAF/ECM evidence supports collagen organization and spatial T-cell exclusion (21–24). Treatment-predictive validity is unestablished (exploratory/mechanistic; candidate pharmacodynamic)	scRNA-seq; scATAC-seq; spatial transcriptomics or digital spatial profiling; mIHC; proteomics	PVTT-specific preclinical rationale for NID1/acarbose; FAP/GJA5–SLIT2/ROBO1 remains mechanistic (12, 13)	External validation, standardized spatial scoring, pharmacodynamic confirmation, safety assessment, and treatment-interaction testing
Myeloid-dominant	C5aR-positive TAMs; SPP1/MMP9 or SPP1/TREM2 states; CCL2/CCR2	Supportive venous-thrombus/metastatic: C5aR-positive TAMs (11) and SPP1/MMP9 or SPP1/TREM2 macrophage states (8, 10); HCC-extrapolated: CCL2/CCR2 (20). PVTT-specific attribution remains limited (exploratory/mechanistic; candidate pharmacodynamic)	scRNA-seq; spatial profiling; mIHC; peripheral inflammatory indices as complementary measures only, not surrogates for tissue-defined myeloid phenotypes	C5aR targeting has thrombus-based biological rationale; CCR2/CSF-1R/TAM-receptor strategies remain extrapolated (11, 19, 20, 52, 53)	Cross-center reproducibility, functional perturbation, and treatment-interaction testing
Hypoxia–adenosine/metabolic	FDG uptake; HIF-1α; CD39/CD73; A2A/A2B; ACLY and fatty-acid programs	PVTT-direct: FDG uptake has PVTT-specific prognostic evidence (36). HCC-extrapolated: HIF-1α, CD39/CD73, A2A/A2B, ACLY, and fatty-acid programs (14, 25–27) (prognostic for FDG uptake; exploratory/mechanistic for pathway biomarkers)	PET/CT; IHC; RNA sequencing; metabolomic or functional assays	Adenosine/metabolic targeting remains HCC-extrapolated; FDG is prognostic, not predictive (14, 25–27, 36)	PVTT-specific pathway activity, predefined cutoffs, and toxicity assessment
Adaptive-checkpoint	TIM-3; LAG-3; VISTA; TOX; TAM-receptor and CD39/CD73 dynamics	HCC-extrapolated: candidate checkpoint and related suppressive programs are derived from primary-HCC or broader studies (4, 14, 19, 37, 38); serial on-treatment PVTT evidence is currently lacking (exploratory/mechanistic; candidate pharmacodynamic)	Paired baseline/on-treatment tissue; mIHC; RNA sequencing; flow cytometry	Alternative-checkpoint strategies remain HCC-extrapolated and unvalidated in PVTT (4, 14, 19, 37, 38)	Paired biopsy studies and evidence of treatment-induced change
Dissemination-associated	CTCs; CTC–white blood cell clusters; ctDNA; extracellular-vesicle cargos	HCC clinical/supportive evidence: CTC phenotypes and CTC–white blood cell clusters (28–30), with broader liquid-biopsy support for ctDNA and related circulating biomarkers (32, 34, 35, 58); direct PVTT-source attribution remains unproven (prognostic/exploratory; candidate longitudinal-monitoring)	Liquid biopsy; ctDNA sequencing; CTC phenotyping; paired phylogenetic analysis	Circulating biomarkers support exploratory monitoring, not treatment selection (28–30, 32, 34, 35, 58)	Source attribution, temporal validation, and incremental value beyond disease burden

None of the candidate biomarkers is currently validated as treatment-predictive in PVTT. References in the “Evidence tier and source” column identify the principal studies supporting each phenotype. Candidate therapeutic implications and priority validation needs represent the authors’ synthesis of the available evidence unless otherwise indicated and should be considered hypothesis-generating. Candidate phenotypes represent literature-informed conceptual groupings rather than empirically derived or validated patient subtypes. Evidence provenance and evidentiary maturity are distinct: PVTT-direct/supportive/HCC-extrapolated describe the source of evidence, whereas established/emerging/hypothesis-generating describe its current level of validation.

## 6 Translational roadmap for PVTT biomarker validation

Translation requires a study architecture that connects anatomical classification with paired tissue, spatial profiling, functional imaging, and liquid biopsy while preserving the distinction between direct and extrapolated evidence (8–13, 29, 30, 36) (**Figure 4**). Throughout this Review, prognostic biomarkers refer to markers associated with clinical outcome irrespective of treatment, predictive biomarkers require evidence of a biomarker-by-treatment interaction, and pharmacodynamic biomarkers refer to treatment-induced biological changes measured longitudinally.

**Figure 4:**
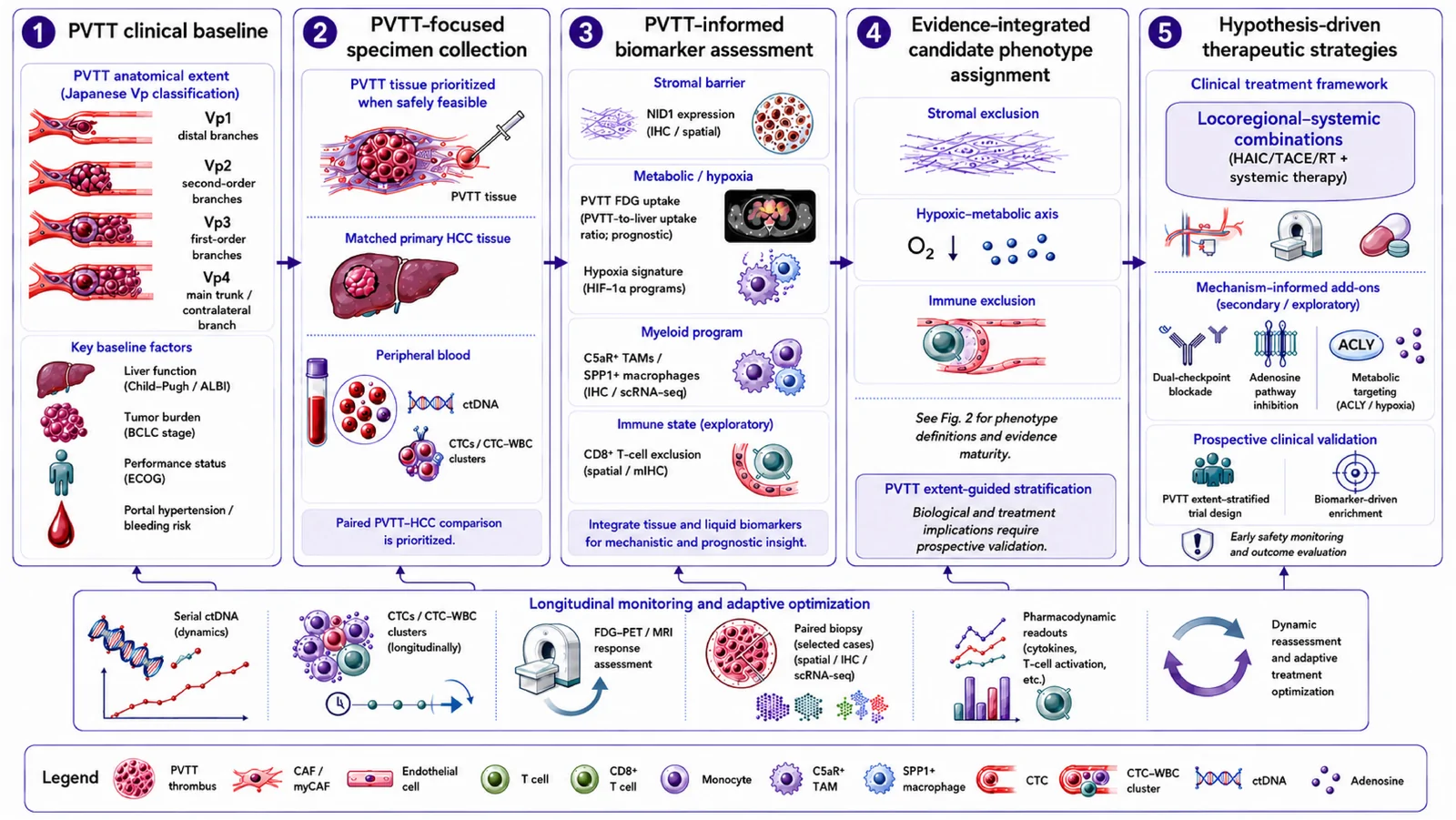
PVTT-specific translational roadmap for biomarker validation and hypothesis-driven therapeutic evaluation. This schematic outlines a PVTT-focused research workflow integrating anatomical stratification, lesion-focused specimen acquisition, multimodal biomarker assessment, evidence-integrated candidate phenotype assignment, and prospective therapeutic evaluation. Clinical baseline assessment incorporates PVTT anatomical extent (Vp1–Vp4), liver function, tumor burden, performance status, and portal-hypertension or bleeding risk. PVTT tissue is prioritized when safely feasible, together with matched primary HCC tissue and peripheral blood, to support anatomically resolved comparisons. Representative PVTT-informed measurements include NID1 expression, the PVTT-to-liver FDG uptake ratio as a prognostic imaging measure, HIF-1α-associated programs, C5aR-positive/SPP1-positive macrophage states, and exploratory CD8-positive T-cell exclusion. These measurements may inform evidence-integrated candidate phenotype assignment and PVTT extent-guided research stratification; phenotype definitions and evidentiary maturity are summarized separately in **Figure 2**. The therapeutic framework distinguishes clinically evaluated locoregional–systemic combinations, including HAIC-, TACE-, or radiotherapy-based combinations with systemic therapy, from secondary and exploratory mechanism-informed approaches such as dual-checkpoint blockade, adenosine-pathway inhibition, and metabolic targeting. Longitudinal assessment using serial ctDNA, CTC/CTC–WBC cluster measurements, FDG-PET or MRI, selected paired biopsies, and pharmacodynamic readouts may support response assessment, early resistance evaluation, and adaptive study procedures. All biomarker assignments and treatment links remain investigational and should not be interpreted as validated clinical treatment-selection rules. ACLY, ATP citrate lyase; ALBI, albumin–bilirubin; BCLC, Barcelona Clinic Liver Cancer; C5aR, complement component 5a receptor; CTC, circulating tumor cell; ctDNA, circulating tumor DNA; ECOG, Eastern Cooperative Oncology Group; FDG, fluorodeoxyglucose; HAIC, hepatic arterial infusion chemotherapy; HCC, hepatocellular carcinoma; HIF-1α, hypoxia-inducible factor 1 alpha; IHC, immunohistochemistry; mIHC, multiplex immunohistochemistry; MRI, magnetic resonance imaging; PET, positron emission tomography; PVTT, portal vein tumor thrombus; RT, radiotherapy; scRNA-seq, single-cell RNA sequencing; SPP1, secreted phosphoprotein 1; TACE, transarterial chemoembolization; WBC, white blood cell.

### 6.1 Paired tissue and spatial validation

PVTT-specific profiling should prioritize matched primary tumor and thrombus specimens, precise anatomical annotation, and sampling of spatially distinct regions. Recent direct studies indicate that myCAF/MMT–NID1 programs and FAP-positive fibroblast/GJA5-positive endothelial hubs can be resolved using combinations of single-cell RNA sequencing, chromatin-accessibility profiling, digital or transcriptomic spatial analysis, and proteomics (12, 13). Discovery features should then be reduced to reproducible, clinically deployable panels with predefined scoring rules. Studies must report whether observations are derived from PVTT alone, mixed venous thrombi, or pooled metastatic compartments.

Validation should address analytical reproducibility, inter-lesion heterogeneity, and clinical transportability. Multicenter cohorts should prespecify sampling procedures, assay platforms, quality-control thresholds, and cutoffs. Prognostic signatures, including reported transcriptomic panels and platelet/CD8-positive T-cell models, require external validation and should not be described as predictive unless a biomarker-by-treatment interaction is demonstrated (33, 54, 55).

### 6.2 Functional imaging and liquid biopsy for longitudinal assessment

Functional imaging may provide a noninvasive measure of biological aggressiveness. The PVTT-to-liver 18F-fluorodeoxyglucose uptake ratio has been associated with progression-free and overall survival, whereas MRI-based immune phenotyping remains unvalidated in PVTT (36, 56, 57). Imaging studies should standardize acquisition, segmentation, normalization, and prespecified cutoffs and should distinguish prognostic information from treatment-predictive value.

Liquid biopsy may complement tissue by tracking total disease burden and temporal change. CTC phenotyping, CTC–white blood cell clusters, ctDNA, and extracellular-vesicle signals warrant longitudinal evaluation, but source attribution remains unresolved (28–30, 32, 34, 35, 58). Changes in these markers should initially serve as exploratory triggers for protocol-defined reassessment, not as stand-alone indications for treatment escalation, de-escalation, or switching.

### 6.3 Biomarker-enriched prospective trials

Future trials should test mechanisms rather than only regimens. Patients should be stratified or enriched according to anatomical extent, liver reserve, portal-hypertension and bleeding risk, tumor burden, and prespecified immune-molecular features. Randomization or biomarker-by-treatment interaction analyses are necessary to distinguish predictive from prognostic effects. Serial tissue sampling in selected patients, functional imaging, ctDNA dynamics, and CTC-related measures can provide pharmacodynamic evidence that a regimen modifies the proposed niche.

Endpoints should extend beyond conventional radiological response to include PVTT-specific response, portal-vein recanalization, conversion to resection, liver-function preservation, bleeding events, quality of life, and dynamic biomarker changes. Adaptive platform designs could connect baseline phenotype assignment with early pharmacodynamic readouts while maintaining prespecified decision rules and independent validation.

## 7 Discussion and future perspectives

The central contribution of this Review is not to redefine macrovascular invasion as a new pathological entity, but to formulate a testable question: does PVTT contain site-dependent tumor–immune programs that are sufficiently reproducible, measurable, and therapeutically relevant to improve stratification beyond anatomy alone? Current evidence supports this proposition only partially. Paired genomic and single-cell studies indicate molecular and ecosystem divergence, thrombus datasets identify suppressive macrophage states, PVTT-specific spatial multi-omics studies have identified myCAF/MMT–NID1 immune barriers and FAP-positive fibroblast/GJA5-positive endothelial hubs, and functional imaging links thrombus metabolism to outcome (8–13, 36). Taken together, the current literature supports three different levels of inference. Anatomical staging, prognosis, and selected treatment outcomes constitute the established clinical context. Clonal/spatial divergence and PVTT-specific stromal programs represent emerging biological evidence that requires independent replication. Hypoxia–adenosine signaling, adaptive checkpoint activation, lipid metabolic remodeling, the PVTT-specific origin of circulating tumor-derived signals, and phenotype-based treatment assignment remain hypothesis-generating and should not be interpreted as established PVTT biology.

The evidence base has important limitations. Most studies are small, cross-sectional, surgically selected, and enriched for patients able to undergo thrombectomy or resection. Some analyses combine PVTT with hepatic-vein tumor thrombus or metastatic lymph nodes, limiting site-specific attribution. Direct on-treatment sampling is rare, and most proposed mechanisms have not been tested with perturbation experiments in PVTT-derived models. These limitations are particularly relevant to extensive Vp3/Vp4 disease, which is clinically important but difficult to sample.

Tissue acquisition itself introduces an additional source of bias. Most PVTT specimens are obtained during surgical resection or thrombectomy, thereby preferentially representing patients with technically accessible and operable disease while underrepresenting unresectable, main-trunk, or treatment-exposed PVTT. Matched primary tumor–PVTT specimens remain limited, and studies based on unpaired lesions may confound true site-specific differences with interpatient heterogeneity. Even when paired samples are available, analysis of one or a few regions may not capture intrathrombus spatial heterogeneity. These constraints highlight the need for anatomically annotated, multiregional paired sampling whenever feasible and for complementary longitudinal imaging or blood-based approaches when repeated tissue acquisition is impractical.

The generalizability of recent PVTT-specific multi-omics findings also remains uncertain. Much of the direct evidence for stromal remodeling derives from a small number of recent studies using surgically accessible or otherwise selected PVTT specimens. These cohorts may underrepresent patients with unresectable, main-trunk, or treatment-exposed disease, and differences in sampling region, assay platform, and analytical pipeline may affect the reproducibility of identified cell states and spatial programs. Accordingly, myCAF/NID1 and FAP/GJA5-associated findings should currently be regarded as strong site-specific signals requiring independent multicenter validation rather than as universally established features of PVTT biology.

The strongest current PVTT-linked signals—clonal divergence, C5aR-positive or related macrophage programs, myCAF/MMT–NID1 and FAP/GJA5 stromal architecture, and high thrombus FDG uptake—remain predominantly descriptive, mechanistic-preclinical, or prognostic. None has demonstrated a reproducible biomarker-by-treatment interaction. CAF/ECM remodeling should therefore no longer be described as wholly extrapolated from primary HCC, but its cross-center reproducibility, assay standardization, and predictive value remain unestablished. Tertiary lymphoid organization, hypoxia–adenosine signaling, adaptive checkpoint activation, and lipid metabolic rewiring remain less directly supported in PVTT. Tissue, imaging, and blood biomarkers should not be treated as interchangeable: circulating signals may reflect total disease burden, whereas anatomically resolved tissue is required to establish local pathway activity and source attribution.

A useful framework must also be falsifiable. The niche model would be weakened if large paired cohorts show no reproducible molecular or spatial divergence between primary HCC and PVTT, if proposed phenotypes cannot be measured consistently across platforms and centers, or if biomarker-defined groups fail to modify treatment effects. The near-term goal should therefore be a layered model in which anatomical extent and liver reserve remain the clinical foundation, while a limited set of reproducible tissue, imaging, and circulating measurements is evaluated prospectively. Clinical adoption should await standardized assays, external validation, longitudinal pharmacodynamic confirmation, and evidence that a biomarker changes treatment benefit rather than merely correlating with outcome.

## 8 Conclusion

PVTT is conventionally defined by anatomical extent, yet paired genomic, single-cell, spatial, imaging, and clinical studies suggest that its biology may not be fully represented by the primary tumor. We therefore propose PVTT as an anatomically distinct, potentially biomarker-definable intravascular tumor–immune niche for hypothesis generation rather than as an established autonomous immune compartment. Current PVTT-direct evidence is strongest for clonal and spatial divergence and PVTT-specific stromal remodeling, including myCAF/NID1 and FAP/GJA5 programs, whereas venous-thrombus studies provide supportive evidence for macrophage-associated immune suppression. By contrast, hypoxia–adenosine signaling, adaptive checkpoint activation, and lipid metabolic rewiring remain largely extrapolated candidate mechanisms that require direct validation in anatomically resolved PVTT samples. The proposed phenotypes should therefore guide mechanistic testing, assay development, and biomarker-enriched trial design rather than routine treatment assignment. Progress will require paired primary tumor–PVTT sampling, standardized spatial and molecular assays, longitudinal pharmacodynamic assessment, and biomarker-by-treatment interaction analyses to determine whether this niche framework can ultimately provide clinically useful stratification.

## Supplementary material

Supplementary Table 1 Study-level summary of principal PVTT-focused biological, biomarker, imaging, and clinical studies included in this Review.
