# Supplementary material for: Immune escape in portal vein tumor thrombus: an intravascular tumor–immune niche framework for hepatocellular carcinoma
Source: Front Immunol. 2026 Sep 16;17:1946103. doi: 10.3389/fimmu.2026.1946103 (PMC13624743; doi:10.3389/fimmu.2026.1946103)
Supplement: Supplementary file 1 [file SupplementaryFile1.docx]

**Supplementary Table S1. Study-level summary of principal PVTT-focused biological, biomarker, imaging, and clinical studies included in this Review**

| **Study** | **Design/population and sample size** | **Specimens or intervention** | **Approach/endpoints** | **PVTT classification** | **Major findings or outcomes** | **Evidence status/current role** |
| --- | --- | --- | --- | --- | --- | --- |
| **A. Tissue-based molecular and multi-omics studies** | | | | | | |
| Su et al., 2021 (9) | Single-cell evolutionary study; 5 liver-cancer patients, including 1 HCC patient with paired primary tumor and intrahepatic PVTT | Primary HCC and paired PVTT | Single-cell mutation profiling and scRNA-seq | NR | Primary HCC and PVTT shared a common origin, followed by early metastatic seeding and independent evolution. | PVTT-direct; emerging |
| Qiu et al., 2021 (54) | Exploratory transcriptomic study; 3 surgically treated HCC patients; 9 matched samples | Normal liver, primary HCC, and PVTT | Bulk RNA-seq and patient-level differential-expression analysis | NR | Substantial interpatient heterogeneity was observed in PVTT-associated transcriptional programs. | PVTT-direct; emerging |
| Zhou et al., 2025 (11) | Integrated immune-profiling study; 249 HCC patients overall; 8-patient discovery cohort plus validation cohorts | Venous tumor thrombus/PVTT, primary tumor, adjacent liver, and peripheral blood | Bulk RNA-seq, WES, CyTOF, scRNA-seq, mIF, and functional analyses | NR | C5aR-positive TAMs were enriched in venous tumor thrombi and linked to suppression of CD8-positive T-cell effector function and adverse outcome. | Supportive venous-thrombus evidence; emerging |
| Han et al., 2026 (12) | PVTT multi-omics study; 47 patients; 99 specimens | Primary HCC/PVTT specimens and spatially annotated tissue regions | nCounter, scRNA-seq, digital spatial profiling, proteomics, and functional experiments | NR | myCAF enrichment, macrophage-to-myofibroblast transition, and NID1-rich immune barriers were identified; acarbose disrupted this stromal program in preclinical models. | PVTT-direct; emerging with preclinical therapeutic evidence |
| Chen et al., 2026 (13) | Single-cell multi-omics discovery study; 10 patients | Multi-region paratumor, tumor-interface, primary HCC, and venous tumor-thrombus specimens | scRNA-seq, scATAC-seq, spatial transcriptomics, and functional assays | NR | FAP-positive fibroblast/GJA5-positive endothelial hubs and SLIT2/ROBO1-related stromal crosstalk were implicated in venous tumor-thrombus progression. | PVTT/venous-thrombus direct; emerging |
| **B. Imaging and prognostic biomarker studies** | | | | | | |
| Lee et al., 2017 (36) | Retrospective imaging cohort; 166 HCC patients with PVTT and no extrahepatic metastasis | Imaging-defined PVTT | FDG-PET/CT; PVTT-to-liver uptake ratio; survival analysis | NR | Higher PVTT FDG uptake was independently associated with shorter progression-free and overall survival. | PVTT-specific prognostic association; not treatment-predictive |
| Shi et al., 2024 (33) | Retrospective prognostic-modeling study; 932 HCC-PVTT patients (training, 656; validation, 276) | Peripheral blood and clinical variables | Platelet/CD8-positive T-cell-based nomogram development and validation | NR | A platelet/CD8-positive T-cell model stratified prognosis in HCC-PVTT; independent external validation remains required. | PVTT-specific prognostic model; emerging, not predictive |
| Tong et al., 2024 (55) | Secondary analysis of public PVTT scRNA-seq datasets plus TCGA/ICGC bulk cohorts | Public primary HCC/PVTT and bulk-HCC datasets | scRNA-seq integration, bulk transcriptomics, and computational prognostic modeling | NA/NR | Immune-desert/exclusion-related programs were nominated and incorporated into an exploratory prognostic model. | Dataset-based; hypothesis-generating |
| **C. PVTT-dedicated clinical treatment studies** | | | | | | |
| Hu et al., 2023 (40) | Multicenter, open-label, noncomparative randomized trial; 60 systemic-treatment-naive HCC-PVTT patients | Camrelizumab plus apatinib with or without SBRT | Overall survival, progression-free survival, response, and safety | Cheng types II-IV | With versus without SBRT, median OS was 12.7 versus 8.6 months, median PFS 4.6 versus 2.5 months, and ORR 47.5% versus 20.0%. | Prospective clinical activity; immune-modulating mechanism unproven |
| Li et al., 2023 (41) | Single-center retrospective real-world cohort; 39 patients | Lenvatinib plus a PD-1 inhibitor and radiotherapy | PVTT response, progression-free survival, overall survival, and safety | Vp4 | PVTT ORR was 61.5%; median PFS was 4.9 months and median OS was 9.4 months. | Retrospective clinical activity; not predictive |
| Li et al., 2024 (42) | Single-center retrospective cohort; 119 patients | HAIC plus anti-PD-1 therapy; TKIs were used in most patients | Response, progression-free survival, overall survival, and safety | PVTT present; subtype distribution NR | ORR was 21.2%; median PFS was 6.9 months and median OS was 14.9 months. | Retrospective clinical activity; immune priming unproven |
| Liu et al., 2024 (43) | Single-center retrospective cohort; 37 patients | mFOLFOX-based HAIC plus TKI and ICI | Volumetric PVTT response, overall survival, and safety | Vp3, n=23; Vp4, n=14 | PVTT ORR was 75.7%; median OS was 15.8 months. | Retrospective clinical activity; not predictive |
| Finn et al., 2024 (50) | Exploratory Vp4 subgroup of global randomized phase III IMbrave150; 73 patients (atezolizumab-bevacizumab, n=48; sorafenib, n=25) | Atezolizumab plus bevacizumab versus sorafenib | Overall survival, progression-free survival, and safety | Vp4 | Median OS was 7.6 versus 5.5 months and median PFS was 5.4 versus 2.8 months; analyses were exploratory. | Established trial context; exploratory subgroup, not biomarker-predictive |
| **Notes:** NR, not reported; NA, not applicable. Evidence status combines evidence provenance with current maturity or clinical role. Clinical activity does not establish immune priming, PVTT-specific causality, or treatment-predictive value unless supported by paired pharmacodynamic evidence or a biomarker-by-treatment interaction. | | | | | | |
